# The LAST Mile: Evaluating Genetic Biocontrol as a Supplemental Tool for Eradicating Invasive Rodents on Islands

**DOI:** 10.1111/eva.70238

**Published:** 2026-04-19

**Authors:** Aysegul Birand, Bruce A. Hay, Matthew Combs, Luke Gierus, Katherine E. Horak, Kevin P. Oh, Louise J. Robertson, Paul Q. Thomas, Ana M. Velasquez‐Escobar, David J. Will, Maciej Maselko, Antoinette J. Piaggio

**Affiliations:** ^1^ School of Biological Sciences University of Adelaide Adelaide Australia; ^2^ Division of Biology and Biological Engineering California Institute of Technology Pasadena California USA; ^3^ United States Department of Agriculture, Animal Plant Health Inspection Service, Wildlife Services National Wildlife Research Center Fort Collins Colorado USA; ^4^ School of Biomedicine and Robinson Research Institute University of Adelaide Adelaide Australia; ^5^ Genome Editing Program, South Australian Health and Medical Research Institute Adelaide Australia; ^6^ CSIRO Health and Biosecurity Canberra Australian Capital Territory Australia; ^7^ Interdisciplinary Graduate Program in Genetics and Genomics, Department of Cell Biology and Genetics Texas A&M University College Station Texas USA; ^8^ Island Conservation Santa Cruz California USA; ^9^ Applied BioSciences Macquarie University Sydney New South Wales Australia

**Keywords:** genetic biocontrol, Gravid Lethal, house mouse, LAST “last mile”, *Mus musculus*, toxicant

## Abstract

Invasive rodents cause severe ecosystem degradation on islands and can be challenging to eradicate. Current best‐practices rely on the application of toxic oral baits and have led to successful eradications and remarkable recoveries of native flora and fauna. Yet this single method is not universally applicable. Genetic biocontrol offers a suite of new solutions to potentially improve outcomes in the critical “last mile” of eradication. These approaches involve the release of genetically modified individuals of the target species to reduce population fitness over time. These include self‐limiting approaches which require multiple releases and self‐sustaining mechanisms (i.e., select gene‐drive systems) which could theoretically collapse populations after a single release. While gene drive systems have received significant attention, their development in vertebrates remains technically challenging, and their ecological and regulatory implications are still in active debate. In contrast, some non‐drive genetic biocontrol approaches, such as Y‐linked editors, fsRIDL, and Gravid Lethal, offer self‐limiting alternatives that may be more immediately deployable. These approaches could be used to supplement toxicant‐based methods and may also have reduced environmental risks and regulatory barriers. To evaluate the potential of these tools, we developed an individual‐based model simulating the eradication of house mice ( *Mus musculus* ) on a 125 ha island with an initial population of 11,000 individuals. We tested various combinations of genetic biocontrol release and effort strategies to understand tradeoffs between required effort and uncertainty for achieving successful eradication. Under certain effort strategies, we found that the Gravid Lethal approach performed the best, achieving eradication in < 2.3 years with intensive release effort and monitoring. Our findings suggest that integrating non‐drive genetic biocontrol into adaptive management frameworks could enhance the effectiveness and feasibility of rodent eradication programs. These tools are not replacements for toxicants but may serve as critical supplements—particularly in the “last mile” of eradication.

## 1 Introduction

Removal of invasive rodents, specifically rats (*Rattus* spp.) and house mice ( *Mus musculus* ), from islands can lead to significant biodiversity recovery and positive trends in overall ecosystem recovery. This recovery contributes to improved land‐sea connectivity, carbon sequestration, nutrient cycling, and island community ecosystem health (Jones et al. 2016; de Wit et al. 2020; Sandin et al. 2022; Graham et al. 2024; Honzák et al. 2024). Over the last 100 years, invasive mammal eradications on islands have increased in complexity and been implemented by a majority of nations with islands (typically small and uninhabited ones), having a success rate of nearly 88% (Spatz et al. 2022). As such, invasive mammal eradications are recognized as one of the most effective conservation practices to prevent extinctions of valued species and halt environmental degradation (Langhammer et al. 2024).

Despite these successes, rodent eradication efforts remain heavily reliant on a single tool: the large‐scale distribution of toxicants. While toxicants have a long and well‐documented history of use in rodent control (Howald et al. 2007; Spatz et al. 2022), and best practices for their application are well established (e.g., Thomas et al. 2017; Broome et al. 2017, 2019; Keitt et al. 2015), their use is not universally feasible. Environmental constraints, non‐target species risks, regulatory barriers, and social acceptability can all limit toxicant application. Moreover, eradication attempts do not always succeed on the first try. Remnant populations may persist due to bait aversion, sublethal exposure, or inaccessible terrain, leading to recolonization and failure to achieve long‐term conservation outcomes (Holmes et al. 2015; Samaniego et al. 2021).

These challenges highlight the need for adaptive management—an iterative process of implementing, monitoring, and adjusting interventions in response to ecological feedback. While adaptive management strategies that rely on a successive series of labor and time‐intensive removal methods to achieve complete removal are commonly used when targeting other invasive species on islands, rodent eradication programs have largely relied on static, single‐method strategies to achieve the overarching set of eradication principles (Cromarty et al. 2002; Parkes 1993). As managers increasingly target larger, more complex islands—including those with human communities—this rigidity limits the effectiveness and scalability of eradication efforts (Harper et al. 2020; Oppel et al. 2011; Glen et al. 2013; Capizzi et al. 2024; Horn et al. 2022). Ambitious landscape‐scale initiatives such as Predator Free 2050 (PF2050) in New Zealand exemplify the growing demand for innovative methods and adaptive management. PF2050 aims to sequentially eliminate multiple invasive mammal species from management zones, including urban areas, using natural features as reinvasion barriers (Parkes et al. 2017; Bell et al. 2019; Patterson et al. 2024). As the size and complexity of target ecosystems increase, so too does the urgency for tool diversification that can support adaptive, multi‐phase eradication strategies. Thus, a range of novel methods should be explored and developed, particularly those that can be used in combination with proven and impactful toxicant interventions (Combs et al. 2023; Campbell et al. 2015; Murphy et al. 2019; Innes et al. 2024).

Innovations, such as the genetic biocontrol strategies discussed below, are unlikely to replace current best practices such as toxicant application but we contend that they may provide valuable synergies when combined with them or other future methods. Most island rodent eradication efforts occur via aerial application of toxicants by helicopter or drone, or through bait stations or hand spreading (Spatz et al. 2022). Successful toxicant‐based rodent eradications sometimes require multiple attempts (MacKay et al. 2007; Holmes et al. 2015; Samaniego et al. 2021; Springer et al. 2024). Due to avoidance of baits, bait interference from invertebrates, and insufficient geographic and topographic coverage (MacKay et al. 2007; Holmes et al. 2015; Samaniego et al. 2021; Kappes et al. 2019). Following an initial application, > 90% of the original population may be removed, but remnant survivors lead to population rebound over weeks to months to pre‐treatment numbers (Hein and Jacob 2015). Upon reviewing these unsuccessful efforts (Springer et al. 2024), there is often not a single defining reason that the implemented operational strategies did not achieve success. Instead, managers are left with several nuanced research questions that can only be addressed through years of research or high‐risk full‐scale operational experimentation to test the efficacy of minor adjustments in strategy.

Current best practices say that if the eradication goals cannot be met with a high degree of confidence through the use of a toxicant, then eradication should be adaptively managed using a series of progressively more resource‐intensive methods to achieve complete elimination of the population. However, for rodent eradications, detecting and eliminating this “last mile” remnant post‐toxicant treatment population remains an enormous challenge (Kappes et al. 2019; Davis et al. 2023). This is particularly true on more complex island systems where features such as difficult terrain, human habitation, and challenging climate create increased opportunities for persistence and rebound of remnant survivors. These points are highlighted in an assessment of recently unsuccessful eradication attempts on the globally important islands of Gough, Tristan da Cunha, and Midway Atoll, United States (Springer et al. 2024). However, recent advances in monitoring technology promise to help more effectively identify remnant rodents (Martinez et al. 2020; Piaggio et al. 2024; Sullivan et al. 2024). These advances, in combination with the use of emerging genetic technologies for biocontrol may provide novel, complementary tools for achieving success in this last mile across island eradication efforts (Piaggio et al. 2017; Teem et al. 2020). Understanding the contexts in which these new genetic biocontrol tools can contribute, and how to optimize combinatorial deployment strategies in the context of rodent behavior and ecology remains a critical step in the effort to increase the success of island eradications (Combs et al. 2023).

Genetic biocontrol comprises a wide range of approaches that aim to suppress a target population following release of organisms with altered genomes (Teem et al. 2020). Genetic biocontrol can be broadly divided into two classes: (1) those which bias their own inheritance, namely gene drives (Burt 2003), which can be self‐sustaining or self‐limiting and; (2) those that are non‐driving, self‐limiting methods that require repeated delivery in order to maintain the genetic element (or edits it creates) in the wild population (Teem et al. 2020). Both classes have the appeal that they leverage natural migration and mating behavior (the desire to seek out conspecifics) to bring about diffusion from the deployment location into the larger target environment—a key distinction from conventional toxicant‐ or trapping‐based approaches. Several synthetic drive strategies have been proposed, and in some cases implemented, for population suppression in insects (reviewed in Bier 2022; Johnson et al. 2024; Beach and Maselko 2025; Tolosana et al. 2025). These positive attributes notwithstanding, modeling suggests that even a relatively invasive gene drive (able to spread relatively rapidly from low frequency), such as the mouse *t*_CRISPR_ system could require a decade or more, if used alone, to eradicate an island population of ~200,000 mice (Birand et al. 2022; Gierus et al. 2022). Thus, while gene drives hold great potential (because the overall effort following initial deployment can be low), the timeframes involved are long and to date have proved inefficient in or non‐transferable to mammals (Grunwald et al. 2019; Pfitzner et al. 2020; Weitzel et al. 2021; Bunting et al. 2024). Furthermore, their potential to impact populations beyond the designated treatment area imposes higher social, cultural, and regulatory barriers.

These facts argue for consideration of other genetic biocontrol strategies in which non gene‐drive strategies that are intrinsically self‐eliminating could be deployed strategically, following an initial population knockdown by toxicants, to increase the likelihood of crossing the last mile from suppression to eradication on islands in which eradication is currently challenging. Non‐driving, self‐limiting genetic biocontrol strategies utilize repeated restocking of individuals carrying the biocontrol element to maintain their effect on the population, such as the highly successful classical biocontrol method, Sterile Insect Technique (SIT) (Knipling 1955; Schwarzländer and Mason 2024). Although they require the release of more individuals than gene drives to suppress the target population, the development times may be shorter and the regulatory, social, and cultural pathway for the use of such elements more acceptable. We cannot predict that non‐self‐sustaining genetic biocontrol would be more socially acceptable than gene drive or toxicants but perhaps using them in combination allows for lower toxicant application and thus synergistic strategies might be more socially acceptable.

Here we consider three transgene‐based (GMO) genetic biocontrol strategies: female‐specific Repressible Inducible Dominant Lethal (fsRIDL), Y‐linked editors, and the Toxic Male Technique (TMT). With fsRIDL, homozygote males are released into the wild (Fu et al. 2007). These carry a tetracycline‐repressible toxin (repressed during rearing) that would be otherwise expressed in female progeny (in environments in which tetracycline is not present), resulting in their death. With repeated releases, the generation over generation decrease in the number of females can lead to population suppression or extinction. A Y‐linked editor consists of a site‐specific DNA‐modifying enzyme such as Cas9 and gRNAs, located on the Y chromosome. These cleave a target sequence on a different chromosome (an autosome or the X chromosome) during adult male spermatogenesis. When these males mate with wild‐type (WT) females the sequence modifications inherited by progeny bring about dominant female‐specific lethality or infertility of progeny. Because the construct acts in the adult male germline to reduce the fitness of female progeny, and the Y‐chromosome is not present in these individuals, the construct is not selected against by the harm it causes. As a result, under ideal conditions (no fitness cost in carrier males), the transgene persists in the population at its introduction frequency. Thus, releases of transgene‐bearing males over multiple generations progressively ratchets the population to a terminal state consisting of fertile males and infertile or dead females (Burt and Deredec 2018). Finally, the idea behind the Toxic Male Technique (TMT) is that males are engineered to produce one or more toxins in their seminal fluid. The hope is that when these are transferred to females during mating, the mated females will die (Beach and Maselko 2025). TMT is different from the first two approaches in that it acts during the release generation. An important corollary is that TMT's suppression ability, unlike that of other strategies (Gierus et al. 2022; Birand et al. 2022), benefits from polyandry, which is common in invasive mice. It could also be very useful for longer lived species where conventional intergenerational transmission may take decades to see an impact. Although TMT is likely to be difficult to engineer in rodents, we reasoned that a TMT—like approach (theoretical at this point) which could rapidly reduce the reproductive capacity of a target population by eliminating mated females may be effective. Here we model a version of TMT called Gravid Lethal. In this system males are engineered to carry a transgene that, when expressed by the placental or embryonic tissues of their offspring, results in the death of the gravid female. This could potentially be achieved via prenatal (embryo‐specific) expression of small molecular weight toxin proteins or enzymes producing toxic secondary metabolites, with the toxin acting non‐autonomously to kill the mother. A drug‐dependent repressible regulatory circuit (e.g., tet‐off) could be used to ensure the transgene is not expressed in lab populations.

For each of the above methods we use modeling to explore if there are genetic biocontrol release efforts and monitoring strategies that could lead to successful eradication when used following application of a toxicant that brings about an initial large decrease in population size (> 90%). The idea is that well‐validated toxicants can be used to rapidly decrease population numbers, thereby providing a context in which modest size releases of non‐driving, self‐limiting genetically modified animals of a single sex (typically males) can be used to bring about final eradication of remnant populations. This approach potentially overcomes the problem of needing to release large numbers of the invasive species required by an unassisted self‐limiting genetic biocontrol approach or releasing animals carrying a self‐sustaining gene drive element. Further, it may lead to broader use applications when toxicant alone could not achieve the last mile or the ability to avoid multiple rounds of toxicant application. We recognize that eradication efforts using genetic biocontrol, specifically self‐limiting based techniques, come with the need for numerous releases of genetically modified animals over long timelines, as compared with large scale aerial toxicant bait applications. However, because some island eradication efforts utilizing only toxicants fail or have other complicating factors (geography, size, a mix of wildlands and human occupation, etc.) we believe it is important to ask if strategies such as genetic biocontrol, which take advantage of the target species' desire to seek out conspecifics for mating, can broaden the possibility for successful eradications to island systems that are currently infeasible. We propose that this approach (LAST; LAst mile Suppression Technology) could fit into the broader pest control paradigm of using a series of methods or tools and adaptive management to achieve eradication.

While the LAST approaches are attractive in principle, to understand contexts in which they could realistically contribute, it is critical to know how many genetic biocontrol individuals would need to be released, how often releases would occur, and what release, monitoring, and management strategies would be most successful. Modeling genetic biocontrol supplementation to toxicant application can allow us to better understand these parameters. With the goal of characterizing parameters and application effort strategies necessary for successful eradication, we use the stochastic, spatially explicit individual‐based modeling framework presented in Birand et al. (2022) to investigate the efficacy of selected genetic biocontrol methods, following an initial toxicant‐mediated reduction in population size, for the eradication of invasive mice, as a rodent model, on islands.

## 2 Methods

### 2.1 The Model

The model is a patch‐based stochastic model where individuals occupy a rectangular array of patches, and each patch holds multiple individuals. However, individuals are not restricted to a single patch but can utilize multiple patches even within a single breeding cycle. Each breeding cycle is considered a time‐step in the model; there are multiple breeding cycles in a year, and generations are overlapping. The following steps occur in each breeding cycle: (1) mate search, (2) mating, (3) reproduction, (4) natal dispersal, (5) survival of adults, and (6) breeding dispersal (for more details, see “The Model” Appendix S1).

#### 2.1.1 Control Strategies

Two control strategies were employed: *lethal control* (toxicant) followed by *genetic biocontrol* (non‐driving). Lethal control was simulated as a range of proportional culling strategies operating on the population. When control was implemented, survival probabilities of all individuals were reduced by multiplier 1—*c*_s_ (reduction in survival with lethal control) per breeding cycle. Subsequently, genetic biocontrol was employed with a release of transgenic males of each of the LAST approaches under varying release strategies with varying effort (see below).

Three genetic biocontrol strategies (LAST) with different assumptions about mating and outcomes were investigated: fsRIDL, Y‐linked editor, and Gravid Lethal. With fsRIDL, WT females mate with transgenic males and the female offspring are transgene‐bearing and suffer mortality, whereas with the Y‐linked editor, the female offspring of the WT females mated with transgenic males are sterile. With the use of Gravid Lethal, the WT females mated with transgenic males suffer mortality without producing any offspring due to expression of a toxic transgene in prenatal development that causes the death of the mother.

#### 2.1.2 Release Strategies for Genetic Biocontrol

**FIGURE 1:**
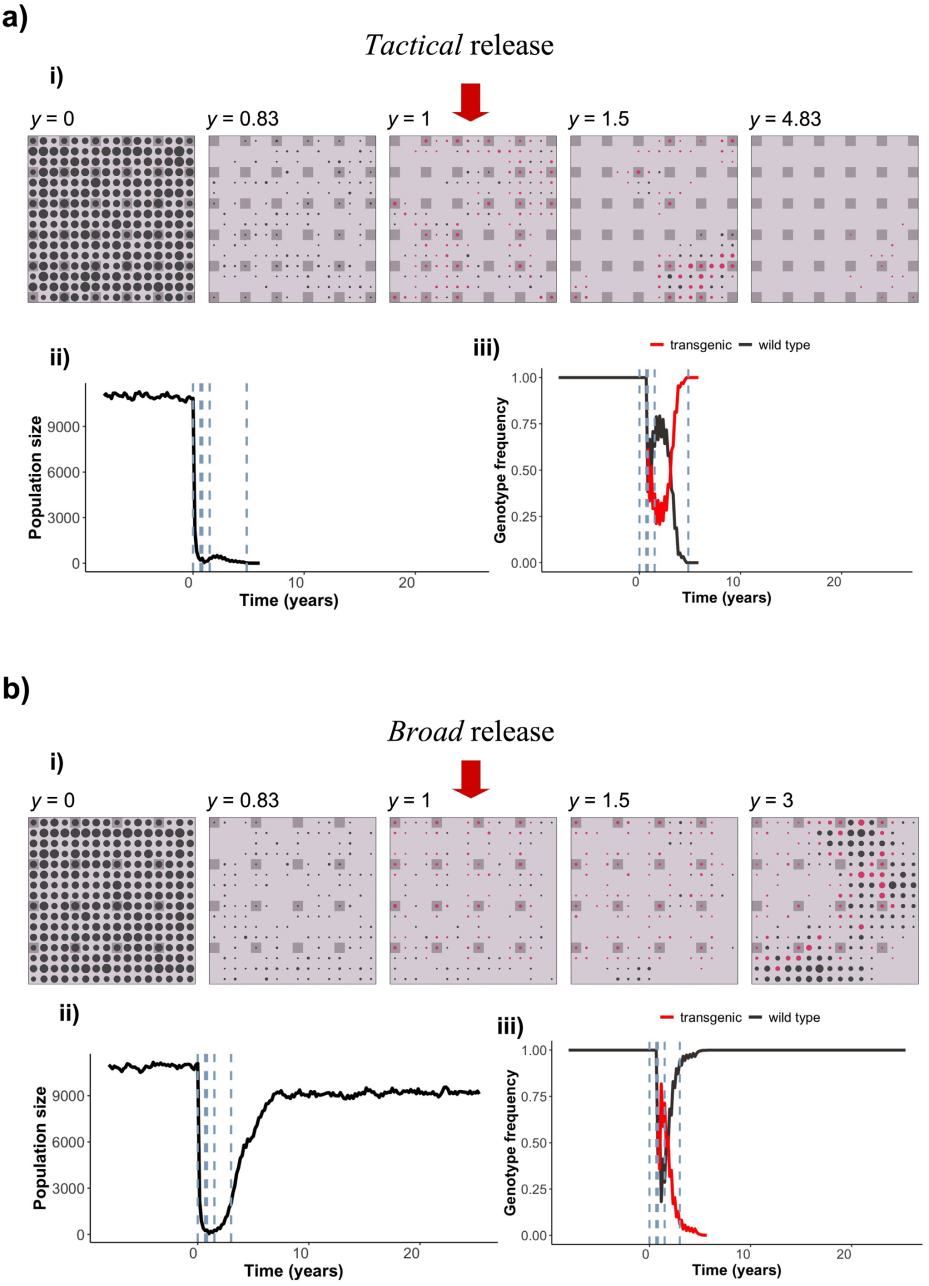
Progression of two sample simulations through time using the Gravid Lethal biocontrol strategy with *tactical* (a) and *broad* (b) release. Each square plot in (i) represents the hypothetical island (as an array of 16 × 16 square patches) with mouse populations through time starting at the end of the eight‐year burn in period (*y* = 0). Populations in patches are represented as circles, the size of which is proportional to the mouse population size in that patch. The black circles represent populations with wild type individuals only; red circles represent populations with one or more transgenic individuals. Dark gray squares are patches where transgenic individuals are released, the same patches are also monitored under *tactical* release in *a*. Empty patches are light gray. The introduction of transgenic individuals started at *y* = 1, after toxicant application l knocked down the population. Released genetic biocontrol individuals can disperse from the release sites before joining the mating pool. (ii) Population size on the island through time. The vertical dashed lines correspond to the times of the simulation snapshots in subplot *i*. (iii) The genotype frequencies of wild type and transgenic individuals through time. Eradication was successful in 6 years in *a*, and failed in *b*. The population size after toxicant application and before genetic biocontrol releases was 39 in *a*, and 42 in *b*. Transgenic mice were introduced to 36 patches in *a*, and 16 patches in *b*. (Other parameters are: *n*_i_ = 12, *N*_i_ = 10, *c*_s_ = 0.20, *D* = 1, *p*_r_ = 0.8, *p*_m_ = 0.8; videos of these simulations are available at https://github.com/abirand/GeneticBiocontrol).

We modeled three release strategies for each genetic biocontrol strategy: *broad*, *tactical*, and *tactical when‐detected*. Under *broad* release, transgenic mice were released to all release sites (*n*_p_). With the *tactical* release strategy, there was monitoring at initial release sites for the presence of individual mice (during the entire release duration, *n*_i_). Transgenic mice were then released only to the sites where mice were detected in the previous breeding cycle (Figure 1). In the final release strategy, *tactical when‐detected*, we assumed that transgenic mice could be differentiated from WT mice during environmental monitoring for DNA (Piaggio et al. 2024). Under this release strategy, releases occurred only if WT individuals were detected, thus reducing the overall release effort and cost to releasing introduction of transgenic mice when possible. Note that each release strategy implicitly assumes variable monitoring efforts, that is, no monitoring effort with the *broad* release, medium monitoring effort the *tactical release*, and high monitoring effort with the *tactical when‐detected* release.

#### 2.1.3 Release Effort

**FIGURE 2:**
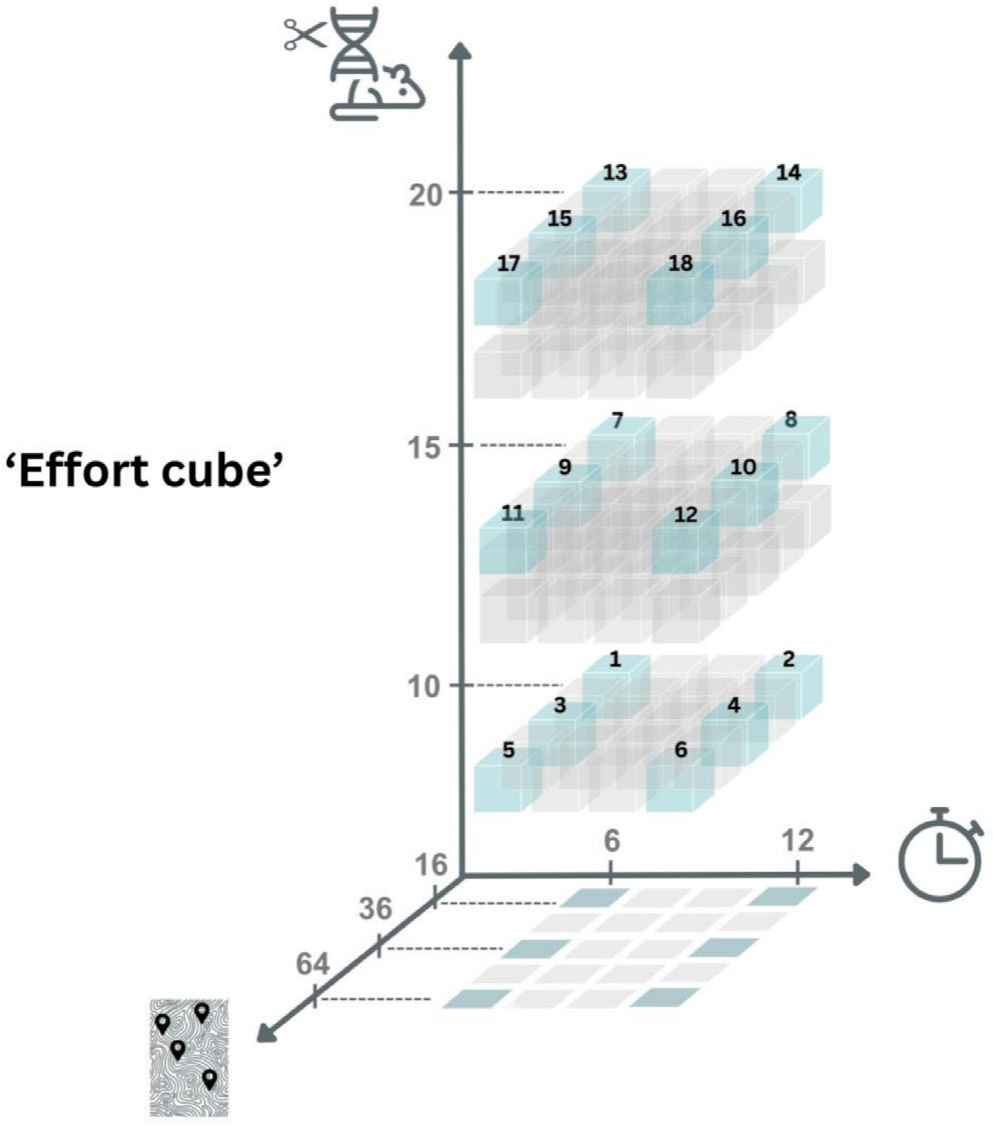
The “effort cube” represents release strategies with varying spatial (*n*_p_, number of introduction sites represented by map graphic), temporal (*n*_i_, number of releases over time represented by clock graphic), and per‐site introduction (*N*_i_, number of transgenic males introduced per site, represented by mouse/DNA/scissors graphic) efforts. Numbers on the axes represent the values used in the simulations (see Appendix S1, Table S1), whereas the numbers on the cubes represent combinations of effort modeled under each release strategy: i.e., #1 represents low spatial effort (*n*_p_ = 16 patches), low temporal effort (*n*_i_ = 6 times) and low per‐site introduction effort (*N*_i_ = 10 transgenic males); whereas #18 represents high effort for all three (64, 12, and 20, respectively). The “shadow” presented in the graph is used to represent the data in Figure 3.

For each release strategy presented above, we varied the overall release effort by changing the spatial effort (*n*_p_, the number of release sites distributed systematically across the island), temporal effort (number of breeding cycles when releases occurred, *n*_i_), and the number of individuals released per patch (*N*_i_) the three dimensional “effort cube” (Figure 2) demonstrates the release combinations investigated in this study. The overall release effort is the total number of transgenic males released, which is *N*_T_ = *N*_i_ *n*_p_ *n*_i_. Note that this definition of effort does not include costs associated with monitoring. In order to accommodate the potential logistical challenges that might occur, we assumed that the releases occurred every other breeding cycle, that is, *n*_i_ = 6 corresponds to 2 years since we assume that there are 6 breeding cycles (*n*_c_) in a year. After being released, for all strategies, transgenic males randomly choose a patch within distance *D* from their introduction sites and join the pool of available males for mating. Females (with probability *p*_r_) choose males randomly among all the available males within the patch, which implies that not all females mate, and some transgenic males may not be chosen for mating. We also assume there is no bias towards mating with WT males.

#### 2.1.4 Parameters

To reduce the parameter space for efficient simulations, we selected mate search and dispersal distances (*D*), levels of polyandrous mating (*p*_m_), and proportion of females mating in the population (*p*_r_) as life history parameters with potentially the highest impact on the efficacy of genetic biocontrol (Birand et al. 2022; Gierus et al. 2022) and investigated those in detail. All parameters related to life history traits, control strategies, and release efforts are presented (Appendix S1, Table S1). Since we are interested in the ability of genetic biocontrol to lead to successful eradications upon the failure of toxicant application to achieve complete population eradication, we chose values for *c*_s_ accordingly and used the simulations in which there were still individuals left on the island (Appendix S1, Figure S1). All release effort combinations with three release strategies for each genetic biocontrol strategy and variable life history traits resulted in 1296 parameter combinations, and we ran 30 simulations for each combination (116,640 simulations in total). Release strategies that vary the combinations of these efforts (spatial, temporal, and introduction size; Figure 2) require different degrees of overall effort and represent trade‐offs that are likely to be important in different environmental and management contexts when toxicant application alone is not successful. Furthermore, we performed a global sensitivity analysis to investigate the relative influence of these parameters on the probability of successful eradication (for more details, see “The Model” and Appendix S1, Table S1). The effects of life history parameters that are not included in the sensitivity analysis were investigated in detail for self‐sustaining genetic biocontrol strategies previously (Birand et al. 2022; Gierus et al. 2022).

#### 2.1.5 Initial Conditions

We assumed that the hypothetical island was 16 × 16 = 256 patches in the model. Each patch corresponds to a 70 m × 70 m space on a hypothetical island of approximately 125 ha (ha). The maximum dispersal distances *D* = [1, 3] in the model correspond to 140–280 m in the wild. The probability of moving these distances increases (see Step 4 in the model, Appendix S1) when the population densities are low; individuals are more likely to remain in their patches at densities close to carrying capacity. We assumed that the population size was ~11,000 before toxicant application. We ran simulations for 200 breeding cycles (33.3 years), which had an initial burn‐in period of 8 years where no control was applied, and a subsequent 1‐year period during which lethal control was simulated as a range of proportional culling strategies (*c*_s_ = 0.15, 0.2) operating on the population. Following the toxicant application, genetic biocontrol males were released (for sample simulations demonstrating release strategies, see Figure 1). We labeled simulation outcomes as unsuccessful if eradication did not occur within the number of breeding cycles simulated. The model is coded using C programming language and is available at GitHub repository https://github.com/abirand/GeneticBiocontrol.

## 3 Results

**FIGURE 3:**
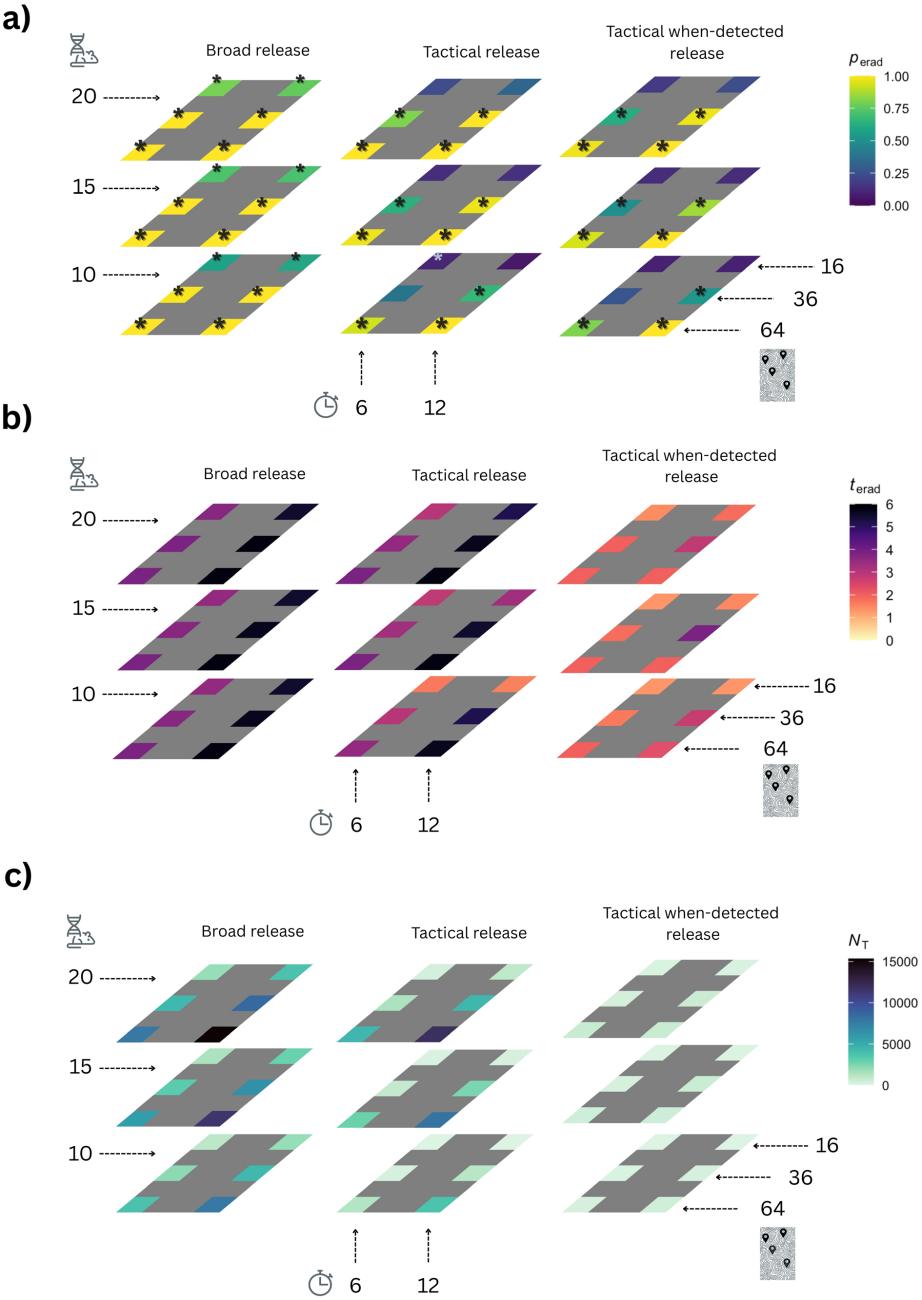
Effort cube for Gravid Lethal genetic biocontrol (a). The probability of eradication for each release effort combination (#1 to #18 in Figure 1) under *broad*, *tactical*, and *tactical when‐detected* release strategies. Note that the mouse/DNA/scissors graphic represents *N*_i_ (number of transgenic males introduced per site) clock graphic represents *n*_i_ (number of releases over time), and map graphic represents *n*_p_ (number of introduction sites). The release efforts where Gravid Lethal outperformed other genetic biocontrol strategies are displayed with “*”. (b) The median time to eradication (in years) for simulations when eradications were successful. (c) The median number of transgenic individuals introduced for each effort combination. Note that the darker shades in panels a–c represent less desirable outcomes with either low eradication probabilities, longer times to eradication, or very high release efforts (in terms of total number of genetic biocontrol individuals introduced). The raw data are provided in Tables S2–S4, for all genetic control strategies (Based on 720 simulations for each combination with variable rates of mate‐search and dispersal distance, *D*, female probability of mating, *p*_r_, and probability of polyandry, *p*_m_).

All three LAST strategies (fsRIDL, Y‐linked editor, and Gravid Lethal) caused permanent eradication with some of the high effort release combinations (Figure 3a and Appendix S1, Table S2, Figures S2 and S3), but other combinations failed. Among the genetic biocontrol strategies examined, overall Gravid Lethal outperformed the other LAST strategies, with higher eradication probabilities for more release effort combinations, particularly when the spatial and/or temporal effort was not high (Figure 3). fsRIDL was the worst performing genetic biocontrol strategy out of the three (Appendix S1, Figure S2), potentially because the reduced local population size due to female offspring mortality resulted in higher fertility rates (due to density‐dependent fertility, see Step 3 in the Model, Appendix S1), which increased the possibility of producing WT offspring. Even though both fsRIDL and Y‐linked editor were less effective compared to Gravid Lethal, release efforts affected the probabilities of eradication. Further, the expected times to eradication and the total number of individuals required in releases that resulted in eradications were qualitatively very similar for all genetic biocontrol strategies (compare Figure 3 with Figures S2 and S3, also Tables S2 and S3 in Appendix S1). There were some effort combinations where the Y‐linked editor outperformed Gravid Lethal (Appendix S1, Figure S3). The results for the rest of this section are based on only Gravid Lethal; however, the general trends hold for the other genetic control strategies as well.

*Broad release* (transgenic mice were released to all the predetermined release sites) outperformed *tactical release* (includes additional monitoring and subsequent releases when the presence of mice was detected) with higher probabilities of eradication for more release effort combinations (Figure 3a). Similarly, *tactical release* outperformed the *tactical when‐detected release* strategy (releases occurred only if WT individuals were detected through environmental monitoring for DNA).

However, both *tactical release* strategies (with or without DNA positive detection in the environment) generally had shorter times to successful eradications (Figure 3b). For example, for the highest overall release effort with 20 transgenic mice per patch to 64 patches, 12 times (release effort combination 18, Figure 2) all three releases were successful (with probability of eradication = 1, Appendix S1, Table S3), the median expected times to eradication were 5.83 for both *broad* and *tactical release* strategies, and were 2 years with the *tactical when‐detected release* strategy. This means that eradication was often achieved before the total number of releases was completed (e.g., the expected median time for successful eradications with release effort combinations 6, 12, and 18 was 2–2.33 years, which corresponded to a temporal effort of *n*_i_ = 6,7 releases; see Appendix S1, Table S3). Overall, increasing release effort on all three dimensions resulted in higher eradication success (Figure 3a).

In cases where eradication was successful, the total number of transgenic mice introduced was also much lower with *tactical release*, which was further reduced with the *tactical when‐detected release* strategy. For example, when 10 transgenic mice per patch were released to 64 patches under the *broad release* strategy for 12 release times (release effort combination #6, Figure 2) there were a total of 7680 transgenic mice released. However, only 3730 and 410 transgenic mice were released under the *tactical* and *tactical when‐detected* release strategies, respectively (for all release effort combinations using three genetic biocontrol strategies, see Appendix S1, Table S4).

Spatial effort (*n*_p_, number of places where transgenic mice were introduced) had a strong impact on eradication probabilities for all release strategies (also see Appendix S1, Figure S4, for the relative influence of *n*_p_ on the probability of eradication). Low spatial effort (*n*_p_ = 16, release combinations 1, 2, 7, 8, 13, and 14, Figure 2) always resulted in low eradication probabilities. Increasing temporal effort (*n*_i_, number of breeding cycles when releases occurred), and per‐site introduction effort (*N*_i_, number of mice introduced per site) when spatial effort was low, did not increase the eradication probabilities further, even with the *broad release* strategy (Figure 3a). With moderate spatial effort (*n*_p_ = 36), increasing temporal effort increased eradication probabilities (e.g., compare release strategies 3 and 4, Figure 2). If both the spatial and the temporal release efforts were not extensive due to time and spatial constraints, increasing per‐site introduction effort increased eradication probabilities (e.g., compare release strategies 3 and 9, Figure 2).

All results presented here were pooled across simulations with variable rates for some of the most critical life history parameters on dispersal and mating that have been shown to impact the success of self‐sustaining murine gene drives (Appendix S1, Table S4; Birand et al. 2022; Gierus et al. 2022). Therefore, the results presented here have included some uncertainty related to these parameters and could be considered as ‘general’ parameters for mice. Sensitivity analysis results (Appendix S1, Figure S4) showed that the relative influence of these life history parameters on probability of eradication is low compared to the relative influence of those related to release strategies, and the effectiveness of toxicant application prior to the introduction of genetic biocontrol. Therefore, our modeling results suggest that even when the exact values for some of the life history parameters are unknown, it is possible to achieve eradication with the LAST approach when toxicant application would have failed.

## 4 Discussion

Despite successful eradications with toxicants, their limitations in certain contexts underscore the need for supplementary tools. Incomplete eradications—due to bait aversion, inaccessible terrain, or sublethal exposure—can leave remnant populations that repopulate and continue to degrade native ecosystems (Holmes et al. 2015). The field of genetic biocontrol holds promise (Teem et al. 2020) for providing new tools. These could be used in an adaptive management context to supplement toxicant applications as a LAST approach and thus increase the likelihood of successful rodent eradication in environments previously deemed intractable.

The results of our modeling of a LAST approach for eradicating an invasive mouse island population shows that the Gravid Lethal strategy is promising as a supplementary tool for contexts in which toxicants may not achieve 100% removal alone. While all three genetic biocontrol strategies could lead to successful eradications, Y‐linked editor and fsRIDL generally required the highest release efforts, that is, *broad* releases with high spatial and temporal effort. Gravid Lethal was successful for more release strategies under *broad*, *tactical*, and *tactical when detected*. This can be understood because with Gravid Lethal, WT females mate with transgenic males and suffer mortality without producing any offspring. In contrast, in the other two LAST strategies eradication is achieved via mortality (fsRIDL) or infertility (Y‐linked editor) of female offspring of transgenic males mating with WT female mice. In other words, these two strategies, in which the possibility of a female mating with multiple males, including WT, provided opportunities for creation of WT progeny, which may then also mate with other WT individuals, thereby decreasing the efficacy of the strategy. Although each of the release strategies we modeled could lead to success, they often required high spatial and temporal release efforts (to 64 patches, 12 times). However, the expected median time to eradication with the Gravid Lethal approach using the *tactical when‐detected* release strategy ranged between 2 and 2.33 years (Appendix S1, Table S3), which means that eradication was achieved after 6–7 releases–much less than the 12 releases for the other strategies–which significantly reduced the total number of individuals introduced (410–680 individuals). It's important to recognize that each release strategy implicitly involves different levels of monitoring effort: *broad* release assumes minimal or no monitoring, *tactical* release requires moderate monitoring, and *tactical release when‐detected* demands more intensive monitoring. There is a trade‐off between the effort invested in release and the effort required for monitoring. Increased monitoring effort for 2–3 years could lead to successful eradications with more modest release effort in terms of total number of individuals introduced. If monitoring is not possible, then high release efforts are needed to achieve successful eradications.

The Gravid Lethal approach differs fundamentally because elimination is in a single generation and leaves no genetic trace. The lack of dependence on intergenerational transmission also renders Gravid Lethal much less susceptible to transgene mutations (e.g., rearrangements) that have been shown to lead to population resistance to gene drives, particularly self‐sustaining approaches (Gierus et al. 2022). Further, although not investigated in detail here, the deployment of Gravid Lethal into a relatively small (post‐toxicant) population would likely provide less opportunity for selection of alleles that are resistant to the transgene effect. We haven't explored the impact of fitness in detail here because without having a developed genetic biocontrol and subsequent captive trials we cannot have informed model parameters for fitness impacts. Also, such impacts that may result in failure of gene drive (Xu and Bonsall 2025) may not be as straightforward for the genetic biocontrol and release strategies we explored. For example, the *broad* release strategy saturates the landscape with transgenic males, and time to eradication with that release strategy is often due to presence of transgenic individuals only. Thus, low fitness of transgenic individuals could unexpectedly reduce the expected time to eradication without impacting the probability of eradication (Figure S5). Continued research and development of self‐limiting genetic biocontrol options for invasive species eradication may lead to development of other approaches that allow us to achieve more successes in a shorter time‐period, with the need for fewer transgenic individuals. However, any approach that is based on the creation of transgenic progeny (e.g., fsRIDL and Y‐linked editors) will need to work well in the face of polyandry, which is common in rodents, and acts to defeat suppression through continued creation of wildtype progeny.

As with all wildlife management tools, no single genetic biocontrol approach is optimal for all circumstances. Multiple factors need to be considered. These include the biology and ecology of the target population, the capacity for deployment at scale, and the capability for spatial and temporal control. The power of the modeling presented herein is that it demonstrates ways these efforts can be tailored to specific applications and where failure is predicted to occur if required efforts are not achievable due to practical or logistical reasons (Figure 3). The model demonstrates that spatial effort is important, with introduction of genetic biocontrol mice to many regularly spaced sites across the island being critical for success. Spatial considerations are also important when surviving mice are detected after toxicant application. How subsequent restocking of genetic biocontrol is deployed in the face of these detections has a large influence on success. The insights gained by the modeling in this regard highlight situations of particular concern, such as when parts of an island are inaccessible, unless other methods of delivery can be utilized (e.g., drone or plane). It will be important for managers considering Gravid Lethal or related approaches to consider each factor carefully for their specific use case. For example, with repeated restocking, the total numbers released over the entire course of control effort may exceed the initial population size on the island, and thus there must be tolerance for short‐term impacts of that introduction. If a transient population increase cannot be tolerated in a specific use case, then this tool would not be viable. Further, the rearing and repeated delivery of a high number of genetic biocontrol mice may not be financially feasible for remote regions. Like other tools, the ones presented here may be useful for some use cases but not others.

Risk assessments can be informed by the factors and outcomes modeled in this study, which will allow a careful consideration of the balance between costs, logistics, and outcome uncertainty (Combs et al. 2023). The strategy of *tactical release* and *tactical when‐detected*, releasing only in areas where mice have been detected, reduces the number of mice required to be released on the island considerably. However, this strategy was successful only if the monitoring effort is extensive across space and time. In addition, the *tactical when‐detected strategy* presupposes the existence of detection technologies with high sensitivity and specificity, a field still at a very early stage. The detection of target species from environmental monitoring (eDNA; e.g., water and soil) provides one such approach and has become a useful monitoring tool (Piaggio 2021) but also increases the effort and cost of eradications, a fact that needs to be considered in feasibility discussions. When monitoring after application of genetic biocontrol, an eDNA assay that can differentiate between WT mice and transgenic mice (Piaggio et al. 2024) could reduce the number of genetic biocontrol mice introduced, by reducing release efforts to only sites where WT mice are detected. When this approach is implemented in the model, the total numbers of genetic biocontrol mice as well as effort dedicated to the release of biocontrol animals and time to eradication are significantly reduced.

We also note that while the other genetic biocontrol methods we explored, fsRIDL and Y‐linked editor, did not have as many pathways to success as Gravid Lethal, there were some that achieved eradication (Appendix S1, Table S2, Figures S2 and S3). The effort cube (Figures 2 and 3, Appendix S1, Table S2, Figures S2 and S3) provides a way to understand the trade‐offs to implementation for each supplemental biocontrol method combined with alternative application strategies and is a useful tool for managers tasked with determining plans for successful eradications.

Finally, we emphasize that the modeling we carried out represents an initial step toward predicting outcomes of various genetic biocontrol interventions. To our knowledge, this is the first modeling study to evaluate the integration of non‐drive genetic biocontrol with toxicant‐based knockdown in an adaptive management framework for invasive rodent eradication (LAST approach). While individual‐based models can offer valuable insights, they are inherently limited by uncertainties in rodent biology and ecology—particularly when applied to specific island contexts. Key factors such as island topology, accessibility for biocontrol deployment, genetic connectivity of populations (Oh et al. 2021), as well as breeding ecology and behavioral dynamics (Moro et al. 2018; Godwin et al. 2019), must be characterized in detail to refine predictions and guide implementation. Our framework allows for the incorporation of such site‐specific data as it becomes available, and we have accounted for variability in life history traits known to influence the success of self‐sustaining murine gene drives (Birand et al. 2022). This approach would also be useful for investigating other management options such as release of chemically sterilized males (Kirkpatrick and Turner Jr 1985; Hess et al. 2024).

## 5 Conclusion

Given the dire impacts of invasive rodents on islands, and the well‐documented positive return on investment from their removal, there is a growing demand for new methods that expand the scope of eradication and reduce the risk of failure. To date, most applications of genetic biocontrol for invasive rodent eradication have focused on gene drives or toxicant‐based strategies, often framed as all‐or‐nothing solutions. Our results demonstrate that self‐limiting genetic biocontrol LAST strategies can serve as effective supplementary tools particularly in the “last mile” of eradication, where one strategy alone may fall short. Modeling results suggest that under certain conditions, the Gravid Lethal approach can achieve eradication following a toxicant‐based knockdown. While the required effort is substantial—similar to that of current toxicant‐only strategies—this approach may extend eradication feasibility to more complex or constrained environments. Importantly, it offers a path forward for adaptive, multi‐method eradication frameworks. We encourage continued development and phased testing of genetic biocontrol strategies beyond self‐sustaining or self‐limiting gene drive approaches, with particular attention to self‐limiting, non‐heritable approaches that may face fewer ecological and regulatory barriers. Public engagement, transparent risk assessment, and site‐specific ecological studies will be essential to ensure these tools are deployed responsibly and effectively in support of island restoration (Akbari et al. 2015; National Academies of Sciences, Engineering, and Medicine 2016; Long et al. 2020).

## Funding

This research was supported in part by the U.S. Department of Agriculture, Animal Plant Inspection Service, Wildlife Services, National Wildlife Research Center. The work was supported by a Discovery Grant from the Australian Research Council (DP190102312) awarded to P.T.

## Ethics Statement

There are no IACUC considerations for this study.

## Consent

The authors have nothing to report.

## Conflicts of Interest

The authors declare no conflicts of interest.

## Supporting information


**Appendix S1:** Description of the model and the sensitivity analysis with additional results.

## Data Availability

No data were collected for this study. The model is coded using the C programming language and is available at the GitHub repository https://github.com/abirand/GeneticBiocontrol.

## References

[eva70238-bib-0001] Akbari, O. S. , H. J. Bellen , E. Bier , et al. 2015. “Safeguarding Gene Drive Experiments in the Laboratory.” Science 349, no. 6251: 927–929. 26229113 10.1126/science.aac7932 PMC4692367

[eva70238-bib-0002] Beach, S. J. , and M. Maselko . 2025. “Recombinant Venom Proteins in Insect Seminal Fluid Reduce Female Lifespan.” Nature Communications 16, no. 1: 219. 10.1038/s41467-024-54863-1 PMC11707029 39774598

[eva70238-bib-0003] Bell, P. , H. Nathan , and N. Mulgan . 2019. “Island Eradication Within Large Landscapes: The Remove and Protect Model.” Island Invasives: Scaling Up to Meet the Challenge 2050, no. 62: 604–610.

[eva70238-bib-0004] Bier, E. 2022. “Gene Drives Gaining Speed.” Nature Reviews Genetics 23: 5–22. 10.1038/s41576-021-00386-0 PMC8344398 34363067

[eva70238-bib-0005] Birand, A. , P. Cassey , J. V. Ross , J. C. Russell , P. Thomas , and T. A. Prowse . 2022. “Gene Drives for Vertebrate Pest Control: Realistic Spatial Modelling of Eradication Probabilities and Times for Island Mouse Populations.” Molecular Ecology 31, no. 6: 1907–1923. 35073448 10.1111/mec.16361 PMC9303646

[eva70238-bib-0006] Broome, K. , D. Brown , K. Brown , et al. 2019. “House Mice on Islands: Management and Lessons From New Zealand.” Island Invasives: Scaling Up to Meet the Challenge 62: 100.

[eva70238-bib-0007] Broome, K. , C. Golding , K. Brown , P. Corson , and P. Bell . 2017. Rat Eradication Using Aerial Baiting Current Agreed Best Practice Used in New Zealand. New Zealand Department of Conservation.

[eva70238-bib-0008] Bunting, M. D. , G. I. Godahewa , N. O. McPherson , et al. 2024. “Investigating the Potential of X Chromosome Shredding for Mouse Genetic Biocontrol.” Scientific Reports 14, no. 1: 13466. 38866815 10.1038/s41598-024-63706-4 PMC11169450

[eva70238-bib-0009] Burt, A. 2003. “Site‐Specific Selfish Genes as Tools for the Control and Genetic Engineering of Natural Populations.” Proceedings of the Royal Society of London. Series B: Biological Sciences 270, no. 1518: 921–928. 10.1098/rspb.2002.2319 PMC1691325 12803906

[eva70238-bib-0010] Burt, A. , and A. Deredec . 2018. “Self‐Limiting Population Genetic Control With Sex‐Linked Genome Editors.” Proceedings of the Royal Society B: Biological Sciences 285, no. 1883: 20180776. 10.1098/rspb.2018.0776 PMC6083257 30051868

[eva70238-bib-0011] Campbell, K. J. , J. Beek , C. T. Eason , et al. 2015. “The Next Generation of Rodent Eradications: Innovative Technologies and Tools to Improve Species Specificity and Increase Their Feasibility on Islands.” Biological Conservation 185: 47–58.

[eva70238-bib-0012] Capizzi, D. , P. Sposimo , G. Sozio , et al. 2024. “For Birds and Humans: Challenges and Benefits of Rat Eradication From an Inhabited Island (Ventotene, Central Italy).” Pest Management Science 80, no. 11: 5510–5518. 38151297 10.1002/ps.7947

[eva70238-bib-0013] Combs, M. A. , A. J. Golnar , J. M. Overcash , et al. 2023. “Leveraging Eco‐Evolutionary Models for Gene Drive Risk Assessment.” Trends in Genetics 39, no. 8: 609–623. 37198063 10.1016/j.tig.2023.04.004

[eva70238-bib-0014] Cromarty, P. L. , K. G. Broome , A. Cox , R. A. Empson , W. M. Hutchinson , and I. McFadden . 2002. “Eradication Planning for Invasive Alien Animal Species on Islands–the Approach Developed by the New Zealand Department of Conservation.” In Turning the Tide: The Eradication of Invasive Species, 85–91. IUCN.

[eva70238-bib-0015] Davis, R. A. , P. J. Seddon , M. D. Craig , and J. C. Russell . 2023. “A Review of Methods for Detecting Rats at Low Densities, With Implications for Surveillance.” Biological Invasions 25, no. 12: 3773–3791.

[eva70238-bib-0064] de Wit, L. A. , K. M. Zilliacus , P. Quadri , et al. 2020. “Invasive Vertebrate Eradications on Islands as a Tool for Implementing Global Sustainable Development Goals.” Environmental Conservation 47, no. 3: 139–148.

[eva70238-bib-0017] Fu, G. , K. C. Condon , M. J. Epton , et al. 2007. “Female‐Specific Insect Lethality Engineered Using Alternative Splicing.” Nature Biotechnology 25, no. 3: 353–357. 10.1038/nbt1283 17322873

[eva70238-bib-0018] Gierus, L. , A. Birand , M. D. Bunting , et al. 2022. “Leveraging a Natural Murine Meiotic Drive to Suppress Invasive Populations.” Proceedings of the National Academy of Sciences 119, no. 46: e2213308119. 10.1073/pnas.2213308119 PMC9674240 36346842

[eva70238-bib-0019] Glen, A. S. , R. Atkinson , K. J. Campbell , et al. 2013. “Eradicating Multiple Invasive Species on Inhabited Islands: The Next Big Step in Island Restoration?” Biological Invasions 15: 2589–2603.

[eva70238-bib-0020] Godwin, J. , M. Serr , S. K. Barnhill‐Dilling , et al. 2019. “Rodent Gene Drives for Conservation: Opportunities and Data Needs.” Proceedings of the Royal Society B: Biological Sciences 286, no. 1914: 20191606. 10.1098/rspb.2019.1606 PMC6842857 31690240

[eva70238-bib-0021] Graham, N. A. , C. E. Benkwitt , and H. P. Jones . 2024. “Species Eradication for Ecosystem Restoration.” Current Biology 34, no. 9: R407–R412. 38714173 10.1016/j.cub.2024.02.033

[eva70238-bib-0022] Grunwald, H. A. , V. M. Gantz , G. Poplawski , X. R. S. Xu , E. Bier , and K. L. Cooper . 2019. “Super‐Mendelian Inheritance Mediated by CRISPR–Cas9 in the Female Mouse Germline.” Nature 566, no. 7742: 105–109. 30675057 10.1038/s41586-019-0875-2 PMC6367021

[eva70238-bib-0023] Harper, G. A. , S. Pahor , and D. Birch . 2020. “The Lord Howe Island Rodent Eradication: Lessons Learnt From an Inhabited Island.” In *Proceedings of the Vertebrate Pest Conference*, 29(29).

[eva70238-bib-0024] Hein, S. , and J. Jacob . 2015. “Recovery of Small Rodent Populations After Population Collapse.” Wildlife Research 42, no. 2: 108–118.

[eva70238-bib-0025] Hess, R. A. , C. J. Park , S. Soto , et al. 2024. “Male Animal Sterilization: History, Current Practices, and Potential Methods for Replacing Castration.” Frontiers in Veterinary Science 11: 1409386. 39027909 10.3389/fvets.2024.1409386 PMC11255590

[eva70238-bib-0026] Holmes, N. D. , R. Griffiths , M. Pott , et al. 2015. “Factors Associated With Rodent Eradication Failure.” Biological Conservation 185: 8–16.

[eva70238-bib-0027] Honzák, M. , G. Roberts , B. J. Cosentino , et al. 2024. “Toward the Quantification of the Climate Co‐Benefits of Invasive Mammal Eradication on Islands: A Scalable Framework for Restoration Monitoring.” Environmental Research Letters 19, no. 11: 114018.

[eva70238-bib-0028] Horn, S. R. , R. L. Sagar , V. K. Frank , et al. 2022. “The Next Frontier: Assessing the Feasibility of Eradicating Mammalian Pests From Auckland Island.” New Zealand Journal of Ecology 46, no. 3: 3500.

[eva70238-bib-0029] Howald, G. , C. J. Donlan , J. P. Galvan , et al. 2007. “Invasive Rodent Eradication on Islands.” Conservation Biology 21, no. 5: 1258–1268. 17883491 10.1111/j.1523-1739.2007.00755.x

[eva70238-bib-0030] Innes, J. G. , G. Norbury , A. Samaniego , S. Walker , and D. J. Wilson . 2024. “Rodent Management in Aotearoa New Zealand: Approaches and Challenges to Landscape‐Scale Control.” Integrative Zoology 19, no. 1: 8–26. 36920845 10.1111/1749-4877.12719

[eva70238-bib-0031] Johnson, M. L. , B. A. Hay , and M. Maselko . 2024. “Altering Traits and Fates of Wild Populations With Mendelian DNA Sequence Modifying Allele Sails.” Nature Communications 15, no. 1: 6665. 10.1038/s41467-024-50992-9 PMC11322531 39138152

[eva70238-bib-0032] Jones, H. P. , N. D. Holmes , S. H. Butchart , et al. 2016. “Invasive Mammal Eradication on Islands Results in Substantial Conservation Gains.” Proceedings of the National Academy of Sciences 113, no. 15: 4033–4038. 10.1073/pnas.1521179113 PMC4839448 27001852

[eva70238-bib-0033] Kappes, P. J. , A. L. Bond , J. C. Russell , and R. M. Wanless . 2019. “Diagnosing and Responding to Causes of Failure to Eradicate Invasive Rodents.” Biological Invasions 21, no. 7: 2247–2254.

[eva70238-bib-0034] Keitt, B. , R. Griffiths , S. Boudjelas , et al. 2015. “Best Practice Guidelines for Rat Eradication on Tropical Islands.” Biological Conservation 185: 17–26.

[eva70238-bib-0035] Kirkpatrick, J. F. , and J. W. Turner Jr. 1985. “Chemical Fertility Control and Wildlife Management.” Bioscience 35, no. 8: 485–491.

[eva70238-bib-0036] Knipling, E. F. 1955. “Possibilities of Insect Control or Eradication Through the Use of Sexually Sterile Males.” Journal of Economic Entomology 48: 459–462.

[eva70238-bib-0037] Langhammer, P. F. , J. W. Bull , J. E. Bicknell , et al. 2024. “The Positive Impact of Conservation Action.” Science 384, no. 6694: 453–458. 38662833 10.1126/science.adj6598

[eva70238-bib-0038] Long, K. C. , L. Alphey , G. J. Annas , et al. 2020. “Core Commitments for Field Trials of Gene Drive Organisms.” Science 370, no. 6523: 1417–1419. 33335055 10.1126/science.abd1908

[eva70238-bib-0039] MacKay, J. W. , J. C. Russell , and E. C. Murphy . 2007. “Eradicating House Mice From Islands: Successes, Failures and the Way Forward.”

[eva70238-bib-0040] Martinez, B. , J. K. Reaser , A. Dehgan , et al. 2020. “Technology Innovation: Advancing Capacities for the Early Detection of and Rapid Response to Invasive Species.” Biological Invasions 22, no. 1: 75–100.

[eva70238-bib-0041] Moro, D. , M. Byrne , M. Kennedy , S. Campbell , and M. Tizard . 2018. “Identifying Knowledge Gaps for Gene Drive Research to Control Invasive Animal Species: The Next CRISPR Step.” Global Ecology and Conservation 13: e00363.

[eva70238-bib-0042] Murphy, E. C. , J. C. Russell , K. G. Broome , G. J. Ryan , and J. E. Dowding . 2019. “Conserving New Zealand's Native Fauna: A Review of Tools Being Developed for the Predator Free 2050 Programme.” Journal of Ornithology 160, no. 3: 883–892.

[eva70238-bib-0043] National Academies of Sciences, Medicine, Division on Earth, Life Studies, Board on Life Sciences, Committee on Gene Drive Research in Non‐Human Organisms and Recommendations for Responsible Conduct . 2016. Gene Drives on the Horizon: Advancing Science, Navigating Uncertainty, and Aligning Research With Public Values. National Academic Press. 27536751

[eva70238-bib-0044] Oh, K. P. , A. B. Shiels , L. Shiels , et al. 2021. “Population Genomics of Invasive Rodents on Islands: Genetic Consequences of Colonization and Prospects for Localized Synthetic Gene Drive.” Evolutionary Applications 14, no. 5: 1421–1435. 34025776 10.1111/eva.13210 PMC8127709

[eva70238-bib-0045] Oppel, S. , B. M. Beaven , M. Bolton , J. Vickery , and T. W. Bodey . 2011. “Eradication of Invasive Mammals on Islands Inhabited by Humans and Domestic Animals.” Conservation Biology 25, no. 2: 232–240. 21054528 10.1111/j.1523-1739.2010.01601.x

[eva70238-bib-0046] Parkes, J. P. 1993. “Feral Goats: Designing Solutions for a Designer Pest.” New Zealand Journal of Ecology 17: 71–83.

[eva70238-bib-0047] Parkes, J. P. , G. Nugent , D. M. Forsyth , et al. 2017. “Past, Present and Two Potential Futures for Managing New Zealand's Mammalian Pests.” New Zealand Journal of Ecology 41, no. 1: 151–161.

[eva70238-bib-0048] Patterson, C. R. , A. Lustig , P. J. Seddon , D. J. Wilson , and Y. van Heezik . 2024. “Eradicating an Invasive Mammal Requires Local Elimination and Reduced Reinvasion From an Urban Source Population.” Ecological Applications 34, no. 3: e2949. 38442922 10.1002/eap.2949

[eva70238-bib-0049] Pfitzner, C. , M. A. White , S. G. Piltz , et al. 2020. “Progress Toward Zygotic and Germline Gene Drives in Mice.” CRISPR Journal 3, no. 5: 388–397. 33095043 10.1089/crispr.2020.0050

[eva70238-bib-0050] Piaggio, A. J. 2021. “Environmental DNA for Conservation.” In Conservation Technology Book, edited by S. Wich and A. K. Piel , 157. Oxford University Press.

[eva70238-bib-0051] Piaggio, A. J. , L. Gierus , D. R. Taylor , et al. 2024. “Building an eDNA Surveillance Toolkit for Invasive Rodents on Islands: Can We Detect Wild‐Type and Gene Drive *Mus musculus* ?” BMC Biology 22: 261. 39548497 10.1186/s12915-024-02063-0 PMC11566076

[eva70238-bib-0052] Piaggio, A. J. , G. Segelbacher , P. J. Seddon , et al. 2017. “Is It Time for Synthetic Biodiversity Conservation?” Trends in Ecology & Evolution 32, no. 2: 97–107. 27871673 10.1016/j.tree.2016.10.016

[eva70238-bib-0053] Samaniego, A. , P. Kappes , K. Broome , et al. 2021. “Factors Leading to Successful Island Rodent Eradications Following Initial Failure.” Conservation Science and Practice 3, no. 6: e404.

[eva70238-bib-0054] Sandin, S. A. , P. A. Becker , C. Becker , et al. 2022. “Harnessing Island–Ocean Connections to Maximize Marine Benefits of Island Conservation.” Proceedings of the National Academy of Sciences 119, no. 51: e2122354119. 10.1073/pnas.2122354119 PMC9907155 36508667

[eva70238-bib-0055] Schwarzländer, M. , and P. G. Mason . 2024. “A Growing Number of Benefit Evaluations and New Innovations Should Foster Broader Adoption of Classical Biological Control.” BioControl 69, no. 3: 215–219.

[eva70238-bib-0056] Spatz, D. R. , N. D. Holmes , D. J. Will , et al. 2022. “The Global Contribution of Invasive Vertebrate Eradication as a Key Island Restoration Tool.” Scientific Reports 12, no. 1: 13391. 35948555 10.1038/s41598-022-14982-5 PMC9365850

[eva70238-bib-0057] Springer, K. , A. Wolfaardt , K. Broome , et al. 2024. “Factors Contributing to Recent House Mouse Eradication Failures on Islands: An Initial Assessment Following a Workshop in New Zealand.” In *Proceedings of the Vertebrate Pest Conference* 31(31).

[eva70238-bib-0058] Sullivan, T. , A. Elzinga , D. J. Will , et al. 2024. “Towards an Automated Camera Trap Monitoring System With Satellite and Drone Upload Capabilities for Island Invasive Mammal Surveillance.” In *Proceedings of the Vertebrate Pest Conference*, 31(31).

[eva70238-bib-0059] Teem, J. L. , L. Alphey , S. Descamps , et al. 2020. “Genetic Biocontrol for Invasive Species.” Frontiers in Bioengineering and Biotechnology 8: 452. 32523938 10.3389/fbioe.2020.00452 PMC7261935

[eva70238-bib-0060] Thomas, S. , K. Varnham , and S. Havery . 2017. “Current Recommended Procedures for UK (Bait Station) Rodent Eradication Projects (Version 4.0).” Sandy Bedfordshire, UK.

[eva70238-bib-0061] Tolosana, I. , K. Willis , M. Gribble , et al. 2025. “AY Chromosome‐Linked Genome Editor for Efficient Population Suppression in the Malaria Vector *Anopheles gambiae* .” Nature Communications 16, no. 1: 206. 10.1038/s41467-024-55391-8 PMC11696527 39747012

[eva70238-bib-0062] Weitzel, A. J. , H. A. Grunwald , C. Weber , et al. 2021. “Meiotic Cas9 Expression Mediates Gene Conversion in the Male and Female Mouse Germline.” PLoS Biology 19, no. 12: e3001478. 34941868 10.1371/journal.pbio.3001478 PMC8699911

[eva70238-bib-0063] Xu, Z. , and M. B. Bonsall . 2025. “Ecology of Gene Drives: The Role of Density‐Dependent Feedbacks on the Efficacy and Dynamics of Two‐Locus Underdominance Gene Drive Systems.” Evolutionary Applications 18, no. 3: e70079. 40059885 10.1111/eva.70079 PMC11885413

